# Central line catheterisation as a cause of vocal cord palsy

**DOI:** 10.1093/jscr/rjaa539

**Published:** 2020-12-28

**Authors:** Emma Richards, Ravinder Suman, Nikoleta Skalidi, Christopher Jennings

**Affiliations:** Ear, Nose and Throat Department, Queen Elizabeth Hosplital Birmingham, Birmingham B15 2TH, United Kingdom; Ear, Nose and Throat Department, Queen Elizabeth Hosplital Birmingham, Birmingham B15 2TH, United Kingdom; Ear, Nose and Throat Department, Queen Elizabeth Hosplital Birmingham, Birmingham B15 2TH, United Kingdom; Ear, Nose and Throat Department, Queen Elizabeth Hosplital Birmingham, Birmingham B15 2TH, United Kingdom

## Abstract

We report an unusual case of vocal cord palsy secondary which developed following insertion of a central line. A 46-year-old gentleman was admitted with seizure activity and reduced GCS. Following failed attempts at establishing intravenous or intraosseous access, a central line was placed into the right internal jugular vein. After extubation, the patient was found to have a right vocal cord palsy. Contemporaneous computed tomography (CT) imaging of the neck and thorax was performed to determine the cause of the palsy. Although this CT was clear, review of the original trauma CT showed a haematoma within the right carotid sheath. This led to a diagnosis of neuropraxia secondary to haematoma from central venous catheterisation. The patient went on to make a full recovery. We discuss our case with review of previous literature and discussion of management in such situations.

## INTRODUCTION

Central line catheterisation is a common procedure. In the USA alone more than five million central venous catheters (CVC) are inserted annually [1]. This can be achieved through the subclavian, or more commonly, through the internal jugular vein (IJV). They are often used for drug administration, haemodynamic monitoring and interventions, and as access for extracorporeal blood circuits [2]. The most common complications associated with central line insertion include cardiac arrhythmias, air emboli, haemothorax, pneumothorax and haematoma [3]. We report a rare case where placement of a CVC into the right IJV resulted in a vocal cord palsy (VCP).

## CASE REPORT

A 46-year-old gentleman was brought in by ambulance in status epilepticus. He was intubated and, following failed attempts to establish intravenous or intraosseous access, a CVC was placed into the right IJV under ultrasound guidance. A trauma series whole body computed tomography (CT) scan was performed after CVC insertion. The patient remained intubated for 9 days until a tracheostomy was performed to help weaning. Four days later it was noted that the patient was unable to phonate despite good airflow past the tracheostomy with a deflated cuff.

He was referred to ENT and flexible nasendoscopy (FNE) showed a right VCP with an immobile, bowed vocal cord (VC) with minimal compensation from the left true and false cords. Fibreoptic endoscopic evaluation of swallowing (FEES) showed no evidence of aspiration.

**Figure 1:**
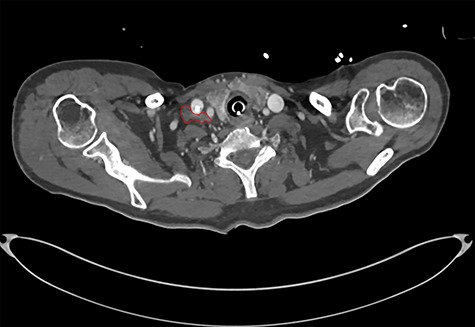
CT showing haematoma in the right carotid sheath following insertion of central line.

**Figure 2:**
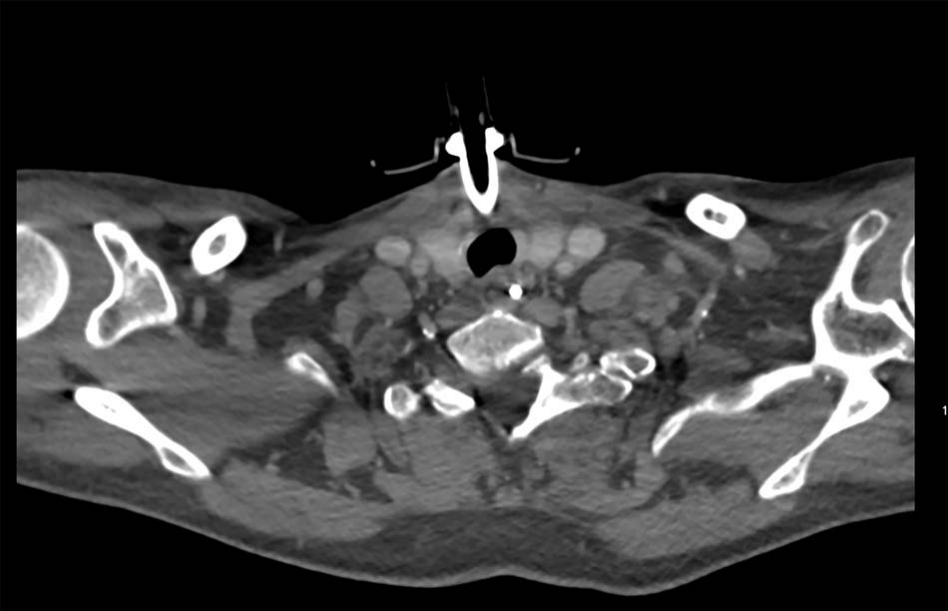
CT showing resolution of the haematoma.

On diagnosing the right VCP, the original trauma series was reviewed by a consultant radiologist who identified a haematoma of the right carotid sheath in the neck (Fig. 1). A second CT scan of the neck and thorax was performed to determine the cause of the VCP 20 days after admission. This did not show a cause for the VCP but did demonstrate resolution of the haematoma (Fig. 2). A second FNE demonstrated resolution of the VCP 29 days following admission.

## DISCUSSION

CVCs can be introduced peripherally, or surgically placed in major neck or subclavian veins, and tunnelled [4]. The right IJV is usually used because of its anatomical continuity with the superior vena cava [5]. Most reported cases of VCP secondary to CVC have involved placement via the IJV [3, 6]. Fishman reported a recurrent laryngeal nerve (RLN) palsy after subclavian line insertion, which is thought to be the first reported case from this site [7].

When investigating our case, CT imaging from skullbase to aortic arch was performed to rule out better known causes of VCP, such as tumours. Differentials considered included palsy secondary to direct trauma from intubation and neuropraxia from cuff pressure. However, the paramedian positioning of the palsied cord supported injury to the RLN, rather than direct trauma [3]. The risk of injury associated with CVC insertion is assumed to be greatest with difficult and repeated attempts [3]. In our case the CVC was placed on the first attempt under ultrasound guidance and only remained in situ for 1 day.

From the limited previous reports of VCP following CVC insertion (Table 1), most have been identified after the development of hoarseness. Bruising is a common feature but only Koduri *et al*. demonstrated a proven haematoma on CT but there was no reported follow-up imaging to show resolution matching recovery of the VCP [1]. Other mechanisms include VCP from local anaesthetic infiltration, with resolution within 3 hours [8].

**Table 1:** Previously reported cases of vocal cord palsy secondary to central line insertion. Central Line Haematoma.

Ref.	Age and Sex	Site	Type of lline	US guidance?	Symptoms	Imaging	Management	Resolution
[1]	77F	IJV	CVC	Yes—seen to puncture common carotid artery	Hoarseness next day	CT—Haematoma	Conservative, SALT	Resolved at 8 month follow-up
[3]	29M	IJV	Hickmann	No—open procedure	Hoarseness next day. Bruising? haematoma	No imaging	Conservative	Died after 2 months
[4]	35 weeks	IJV	CVC	No—open	Inspiratory stridor on extubation	No	Conservative	Resolved at 6 month follow-up
[5]	57F	IJV	CVC—2 attempts	No	Hoarse and coughing when extubated. Bruising at CVC site	None	Conservative	Resolved at 8 month follow-up
[5]	43F	IJV	CVC—2 attempts	No	On removing CVC developed immediate hoarseness	None	Conservative	Resolved at 1 year
[6]	50M	IJV	Chemo port	No	Immediate hoarseness, right neck swelling	None	2 weeks PO steroids	Resolved at 9 month follow-up
[6]	39F	IJV	Chemo port	No	Hoarseness. Seen by ENT after 5 days	None	2 weeks PO steroids	Resolved at 1 month follow-up
[6]	53F	IJV	Chemo port	No	Hoarseness, coughing with water. Seen at 2 weeks by ENT	None	2 weeks PO steroids	Resolved at 1 week follow-up
[6]	76F	IJV	Chemo port	No	Hoarseness on Day 4 postprocedure	None	2 weeks PO steroids	Resolved at 3 weeks follow-up
[7]	63M	Subclavian	Hickmann	Yes	Immediate dysphonia	CT—no cause found	Conservative	Unknown
[8]	47F	IJV	CVC	No	Hoarseness, SOB, stridor	No	Conservative	3 hours, likely secondary to local anaesthetic
[9]	55F	IJV	CVC	No	Hoarseness next day	No	Conservative	Unknown
[10]	58F	IJV	CVC—both sides attempted	No	Visible haematoma and hoarse after first side. Haematoma after second side then needed emergency tracheostomy	No	Emergency tracheostomy	Recovered at 2 weeks

Fishman suggested that the cause of the VCP can be indicated by: the timing of events, whether it is unilateral and ipsilateral to site of line insertion, and whether the voice fails to improve within the time expected for an intubation-related injury [7]. Taking into account these factors, the clinical history, haematoma seen on the original CT, and lack of any other likely cause, the reason for the VCP was determined as neuropraxia secondary to haematoma from insertion of the central line. This reasoning was further supported by the fact that the onset and recovery of the VCP, mirrored the development and resolution of the haematoma. As, at the time of referral, the central line had been removed, this could easily have been missed as a potential cause of VCP and highlights the importance of a good history.

Given the increasing recognition of this potential risk, even when ultrasound guidance is used, Salman *et al*. recommended a pre-insertion VC check by the anaesthetist in suitable cases [3]. Similarly, appropriate justification and counselling is needed if performing CVC on the contralateral side for a patient with known VCP [9]. For patients undergoing surgery where the RLN is felt to be at risk, Martin-Hirsch *et al*. advocated ipsilateral placement of the central line where possible. This is due to the increased morbidity and severe consequences of a bilateral VCP, including need for tracheostomy [5]. As illustrated by Butsch *et al*., if hoarseness develops after difficult attempts to catheterise, further attempts should not be made on the contralateral side, unless absolutely necessary. In their case, where catheterisation of the second side was attempted, the patient developed dyspnoea and bilateral VC paralysis necessitating tracheostomy [10].

If a VCP is found which is thought to be secondary to a haematoma from central line placement we recommend observation and supportive therapy in the first instance. This is supported by the time frame for recovery seen in previously reported cases (Table 1). Our patients’ palsy recovered 1 month following CVC insertion. Salman *et al*. also stated that any remedial intervention should be delayed for at least 12 months because late recovery can occur [3]. VC injection should be avoided unless the palsy is prolonged and then use of a temporary agent such as fat or hyaluronic acid is appropriate given the likelihood of recovery. Speech and language team involvement to help improve swallowing function is recommended.

This case highlights that VCP is a rare, but increasingly recognised, potential complication of CVC insertion. We found no other case where CT imaging of haematoma presence and resolution was clinically matched with resolution of the VCP. A thorough history and careful interpretation of examination findings and imaging are integral to facilitating diagnosis and appropriate management.
